# Galerkin finite element analysis for magnetized radiative-reactive Walters-B nanofluid with motile microorganisms on a Riga plate

**DOI:** 10.1038/s41598-022-21805-0

**Published:** 2022-10-27

**Authors:** Faisal Shahzad, Wasim Jamshed, Usman, Rabha W. Ibrahim, Farheen Aslam, El Sayed M. Tag El Din, Hamiden Abd El-Wahed Khalifa, Fayza Abdel Aziz ElSeabee

**Affiliations:** 1 Department of Mathematics, Capital University of Science and Technology (CUST), Islamabad, 44000 Pakistan; 2 Department of Computer Science, National University of Sciences and Technology, Balochistan Campus (NBC), Quetta, 87300 Pakistan; 3 Near East University, Mathematics Research Center, Department of Mathematics, Near East Boulevard, Nicosia/Mersin 10, PC: 99138 Turkey; 4 Department of Biotechnology, Lahore College for Women University, Lahore, 54000 Pakistan; 5 Electrical Engineering, Faculty of Engineering and Technology, Future University in Egypt, New Cairo, 11835 Egypt; 6 Department of Operations Research, Faculty of Graduate Studies for Statistical Research, Cairo University, Giza, 12613 Egypt; 7 Department of Mathematics, College of Science and Arts, Al-Badaya, Qassim University, Buraydah, 51951 Saudi Arabia; 8 Mathematics Department, Faculty of Science, Helwan University, Cairo, Egypt; 9 Department of Mathematics, College of Science and Arts, Alasyah, Qassim University, Buraydah, Saudi Arabia

**Keywords:** Mathematics and computing, Physics

## Abstract

In order to understand the characteristics of bio-convection and moving microorganisms in flows of magnetized Walters-B nano-liquid, we developed a model employing Riga plate with stretchy sheet. The Buongiorno phenomenon is likewise employed to describe nano-liquid motion in the Walters-B fluid. Expending correspondence transformations, the partial differential equation (PDE) control system has been transformed into an ordinary differential equation (ODE) control system. The COMSOL program is used to generate mathematical answers for non-linear equations by employing the Galerkin finite element strategy (G-FEM). Utilizing logical and graphical metrics, temperature, velocity, and microbe analysis are all studied. Various estimates of well-known physical features are taken into account while calculating nanoparticle concentrations. It is demonstrated that this model's computations directly relate the temperature field to the current Biot number and parameter of the Walters-B fluid. The temperature field is increased to increase the approximations of the current Biot number and parameter of the Walters-B fluid.

## Introduction

Nanofluids (NFs) are being considered as a viable liquid replacement that might raise the ability and effectiveness of current systems in manufacturing, commercial, and residential settings. Developed thermal system efficiency has many advantages, including lessening the negative effects on the environment, using less energy, and spending less money. The cost and environmental effect of NFs were assessed using sustainability methods to evaluate whether they were appropriate for use in thermal systems. Studies of heat are among its most important uses. Thermal systems' energy utilization is essential to the global ecology. Increased heat surface area is one of the most investigated methods for improving thermal exchangers' thermal presentation; yet, this modification results in significant accumulation and drives up manufacturing costs. Recent developments in solar collectors and the usage of NF were discussed by Gupta et al.^1^. They discovered that the most effective strategy to boost the performance of a solar energy system is to employ a premium heat transfer fluid with remarkable thermophysical characteristics, such as high thermal conductivity, and NF is the best option for doing so. By lowering the heat of the liquid leaving the condenser, according to Salilih et al.^2^, the use of NF boosted the effectiveness of the solar scheme. Olabi et al.^3^ conducted research on waste heat recovery, or the process of recovering energy losses as heat, work, or power. They asserted that the high-performance heat transfer (HT) fluids known as NFs had only recently been created. It is recommended that heat transfer development or inhibition as well as the usage of NFs be applied to various types of temperature pipes in accordance with the hypothesis advanced by Wang et al.^4^. The application of machine learning is explored in NFs (thermal conductivity and dynamic viscosity) and NF-charged heat pipes. Souza et al.^5^ looked at the most current advancements in NF thermal properties and applications in a range of engineering sectors, from NF-medicine to renewable energy. The latter has seen some significant improvements in mobility and propulsion, with ramifications for military and defense technologies. In a sono heat exchanger, Nugroho et al.^6^ looked at how ultrasonic waves affected the HT and NFs stability of MWCNTs. In their study Reddy et al.^7^ examined the properties of energy and sticky dissipation on the MHD HT of NFs movement over a nonlinear expanding surface with chemical reaction.

The constitutive associations of Maxwell are insufficient to characterize a large number of viscoelastic fluids. Elastico-viscous fluids come in two different varieties: Rivlin-Ericksen and Walters' B fluids model (WBFM). According to Walters^8^, a mixture of polymethyl methacrylate and pyridine with a density of 0.98 g per litre and 30.5 g of polymer per litre performs nearly identically to WBFM at 25 °C. WBFM is utilized to produce a number of necessary polymers and priceless products. The heat particle statement analysis for nonlinear thermally industrialized flow of WBFM with resistance powers was described by Chu et al.^9^. Waqas et al.^10^ explored the reactions in the WBFM with nonlinear heat energy and motivation dynamism bio-convection immobility point slip flow. The heat of ferromagnetic WBFM was highlighted by Siddique et al.^11^ by changing current categorization. The stability physiologies of WBFM in a cylindrical figure with thermal transfer were studied by Awasthi et al.^12^. Ahmad et al.^13^ noted that relative growth for the energy and attentiveness contours was suggested by the non-axisymmetric flow of unstable WBFM over a vertical cylindrical disk. An accurate characterization of the electro-osmotic flow of the WBFM with a non-singular kernel was reported by Sunthrayuth et al.^14^. The unproductivity point of WBFM over power-law filing exterior with slip situations was inspected by Ahmad et al.^15^. Utilizing the guidance of strange recreation analysis, Idowu and Falodun^16^ observed the Soret-Dufour properties on MHD heat and mass transmission of WBFM over a semi-infinite perpendicular disc. For more details see Refs.^17–26^.

An electromagnetic shallow known as a Riga plate (RP) is used to alternatively build electrodes. This setup results in electromagnetic hydrodynamic behavior in the fluid flow. In this investigation, the effects of thermal radiation, melting heat, and viscous dissipation on the RP are examined. Akolade and Tijani^27^ considered RP in a comparative study of three-dimensional flow of Casson–Williamson NFs. Hakeemet al.^28^ presented RP in 3D- viscous dissipative movement of NFs. Rawat et al.^29^ introduced a numerical study of thermal radiation and suction effects on copper and silver water NFs over RP. Rasool and Zhang^30^ considered the RP to characterize the chemical reaction and convective boundary conditions in Powell-Eyring NFs movement. Asogwaet al.^31^ suggested a proportional examination of aquatic constructed Al_2_O_3_ NFs through water created CuO NFs via enhanced radiate RP.

In contrast to heterogeneous reactions (HHRs), which have reactants in two or more phases, homogeneous reactions are chemical processes in which the reactants and products are in the same phase. Heterogeneous reactions are those that happen on the surface of a catalyst made of a dissimilar level. Anuar et al.^32^ considered NFs stagnation point movement via MHD stretching/shrinking sheet in presence of HHRs. Mishra et al.^33^ analyzed HHRs in a NFs past a nonlinear widening shallow. Almutairi et al.^34^ utilized MHD movement of NFs with HHRs in a porous medium further down the effect of second-order velocity slide. Li et. al.^35^ modified the motion of NFs with HHRs. Raeeset al.^36^ suggested a HHR model for assorted convection in gravity-driven film movement of NFs.

A procedure called bioconvection addresses the wave of mobile microorganisms, which might be useful in preventing the likely relaxing of NFs entities. Waqas et al.^37–43^ considered this process in different studies via various types of NFs, such as Carreau–Yasuda, Casson, Buongiorno and Oldroyd-B NFs. Analysis and numerical studies using different mathematical modeling are given by Beg et al.^44^, Sajid et al.^45^, Waqas et al.^46^, Khan et al.^47^ and Imran et al.^48^. Muhammad et al.^49^ presented a study on especially stratified bioconvective transference of Jeffrey NFs with gyrostatic motile microorganisms. Rao et. al.^50^ indicated the bioconvection in a convectional NFs movement containing gyrotactic microorganisms done by an isothermal perpendicular funnel entrenched in an absorbent shallow with chemical reactive type. Recent additions consider nanofluids with heat and mass transfer in various physical situations^51–54^.

The Finite element method (FEM) is a general arithmetical approach for answering PDEs in two or three spatial variables. All mathematical modeling systems, especially those involving mass and heat movement, heavily employ this technique. It first appeared in Ahmad's study^55^, where the goal was to use FEM to simulate the NFs movement and heat arenas inside a motivated geometry that was home to a heat-generating device. Using the generalized FEM, Hiba et al.^56^ optimized hybrid NFs. The melting effect on CCHFM and heat energy types for allied MHD NFs movement was measured by Ali et al.^57^ using the FEM methodology. Using FEM, Abderrahmane et al.^58^ achieved the best result for non-Newtonian NFs. With the aid of FEM, Rana and Gupta^59^ created a solution for the quadratic convective and active movement of hybrid NFs over a rotating pinecone. On a widening shallow of Chamfer flippers, Pasha and Domiri-Ganji^60^ used FEM to study a hybrid NF. In an animated inclusion with switch cylindrical cavity, Redouane et al.^61^ investigated the thermal movement flood of hybrid NFs under the assumption of generalized FEM. Using FEM, Alrowaili et al.^62^ demonstrated a magnetic radioactive convection of NFs. A dynamic system of NFs was modeled by Zaaroura et al.^63^ using a FEM-optimized homogenization technique. Using FEM simulation, Ahmed and Alhazmi^64^ impacted the revolution and various heat conditions of rolls with glass spheres in the presence of radioactivity.

The prior literature demonstrates that the properties of bio-convection and moving microorganisms in flows of magnetized Walters-B nano-liquid with convective border constraints were not before studied. To close this gap in the interpretation, the Walters-B nanofluid and the heat diffusion theory in the presence of Brownian and thermophoretic diffusion were both included. A stretchable sheet with Riga plate was employed in the current experiment to simulate the physical state. This work is very beneficial in many different technological and industrial domains. The higher-order nonlinear ODEs that were produced were solved with COMSOL software to generate G-FEM solutions for nonlinear equations. Graphical representations were used to show the effects of various flow parameters on the velocity, temperature, and concentration of Walters-B nanofluid. Additionally, the Walters-B nanofluid's Nusselt, Sherwood, and consistency quantities were determined and examined.

## Governing equations and material

**Figure 1:**
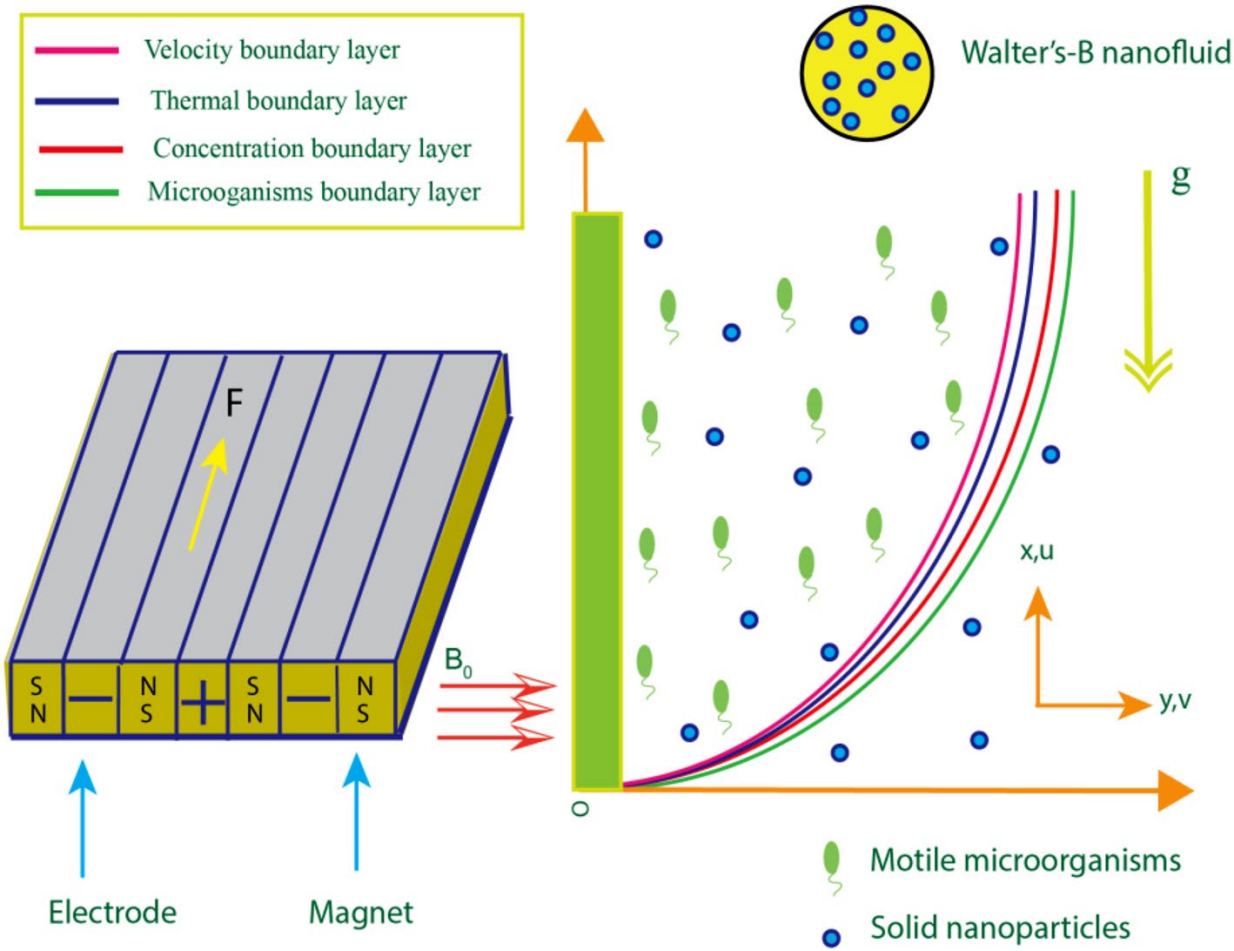
Flow chart for a current problem.

Use a 2D steady flow of magnetized Walters-B nano-liquid across a stretchy surface to examine the characteristics of bio-convection and migrating microorganisms along the fluid flow in x-path. With a stretching speed of u = U w = cx, the xy-coordinate system is utilized, with the x-axis parallel to the flowing direction and the y-axis parallel to the flowing path, as illustrated in Fig. 1. The magnetic field has been subjected to B_0_. The Walters-B nanoliquid is expected to be in a diluted suspension due to a uniform distribution of gyrotactic microorganisms. When suspended nanoparticle concentrations are less than 1%, gyrotactic bacteria' swimming velocity and orientation are unaffected by nanoparticles. It is also hypothesized that when the suspended nanoparticles concentration is low, the bio convection effect is exacerbated. Both the wall's temperature (T_w_) and concentricity (C_w_) are kept constant. There are two cooled extended surface scenarios: T_w_ < T_∞_ and C_w_ < C_∞_. The impacts of activation energy and bilateral reaction are anticipated to be significant due to the study's anticipated high species concentration. Where the width of the magnets is placed in the space between the electrodes, represents the magnetic property of the permanent magnets' plate surface, and represents the amount of current flowing through the electrodes.Where *r*_0_ denotes the width of the magnets positioned in the interval separating the electrodes, *M*_0_ denotes plate surface magnetic property of the permanent magnets, *j*_0_ denotes current density applied to the electrodes.

In view of the proposed assumptions, the governing equations and corresponding boundary conditions for Walters-B nanofluid flowing following^65,66^

1 $$\frac{\partial u}{\partial x}+\frac{\partial v}{\partial y} = 0,$$

2 $$\left.\begin{array}{c}\begin{array}{l}u\frac{\partial u}{\partial x}+v\frac{\partial u}{\partial y}=\nu \frac{{\partial }^{2}u}{\partial {y}^{2}}-\frac{{k}_{0}}{\rho }(u\frac{{\partial }^{3}u}{\partial x\partial {y}^{2}}+v\frac{{\partial }^{3}u}{\partial {y}^{3}}+\frac{\partial u}{\partial x}\frac{{\partial }^{2}u}{\partial {y}^{2}}-\frac{\partial u}{\partial y}\frac{{\partial }^{2}u}{\partial x\partial y})-\frac{\sigma {B}_{0}^{2}}{\rho }(u-{U}_{e})\\ +{U}_{e}\frac{d{U}_{e}}{dx}+\frac{1}{{\rho }_{f}}\left[\left({\rho }_{f}{\beta }^{**}\right){g}^{*}\left(T-{T}_{\infty }\right)-\frac{{M}_{p}\left({\rho }_{p}-{\rho }_{f}\right){g}^{*}}{{\rho }_{p}}\left(C-{C}_{\infty }\right)-\gamma \left({\rho }_{m}-{\rho }_{f}\right){g}^{*}\left(N-{N}_{\infty }\right)\right]\end{array}\\+ \frac{\pi {j}_{0M}}{8\rho }{e}^{-\frac{\pi }{{r}_{0}}y},\end{array}\right\}$$

3 $$\left.\begin{array}{l}u\frac{\partial T}{\partial x}+v\frac{\partial T}{\partial y}=\alpha \frac{{\partial }^{2}T}{\partial {y}^{2}}+\frac{\partial }{\partial y}\left(\frac{16\alpha {\sigma }^{*}{T}_{\infty }^{3}}{3{k}^{*}}\frac{\partial T}{\partial y}\right)+\frac{{Q}_{o}}{\rho {C}_{p}}\left(T-{T}_{\infty }\right)+{\lambda }_{t}^{*}\frac{{Q}_{o}}{\rho {C}_{p}}\left(v\frac{\partial T}{\partial y}+u\frac{\partial T}{\partial x}\right)\\ +{\lambda }_{t}^{*}\tau {D}_{B}\left(u\frac{{\partial }^{2}C}{\partial x\partial y}\frac{\partial T}{\partial y}+u\frac{\partial C}{\partial y}\frac{{\partial }^{2}T}{\partial x\partial y}+v\frac{{\partial }^{2}C}{\partial {y}^{2}}\frac{\partial T}{\partial y}+v\frac{\partial C}{\partial y}\frac{{\partial }^{2}T}{\partial {y}^{2}}\right)\\ +{\lambda }_{t}^{*}\tau \frac{{D}_{T}}{{T}_{\infty }}(\frac{\partial T}{\partial y}{)}^{2}+2{\lambda }_{t}^{*}\tau \frac{{D}_{T}}{{T}_{\infty }}\left[u\frac{\partial T}{\partial y}\frac{{\partial }^{2}T}{\partial {y}^{2}}+v\frac{{\partial }^{2}T}{\partial {y}^{2}}\frac{\partial T}{\partial y}\right],\\ \end{array}\right\}$$

4 $$\left.\begin{array}{l}u \frac{\partial C}{\partial x} + v \frac{\partial C}{\partial y} = {D}_{B}\frac{{\partial }^{2} C}{\partial {y}^{2}}+{\lambda }_{c}^{*}\frac{{D}_{T}}{{T}_{\infty }}(\frac{{\partial }^{2} T}{\partial {y}^{2}} + \frac{\partial v}{\partial y} \frac{{\partial }^{2} T}{\partial {y}^{2}} + \frac{\partial u}{\partial y} \frac{{\partial }^{3} T}{\partial x\partial y})\\ -K{r}^{2} (C - {C}_{\infty })(\frac{T}{{T}_{\infty }}{)}^{n}exp(\frac{-{E}_{a}}{k T}) - {\lambda }_{c}^{*} {k}_{c} ( v \frac{\partial C}{\partial y}+u\frac{\partial C}{\partial x})- {\lambda }_{c}^{*} [{u}^{2} \frac{{\partial }^{2} C}{\partial {x}^{2}} \\ + {v}^{2} \frac{{\partial }^{2} C}{\partial {y}^{2}} + 2 u v \frac{{\partial }^{2} C}{\partial x \partial y} + \frac{\partial C}{\partial x}( u \frac{\partial u}{\partial x} + v \frac{\partial u}{\partial y})+\frac{\partial C}{\partial y}( u \frac{\partial v}{\partial x} + v \frac{\partial v}{\partial y} ) ],\end{array}\right\}$$

5 $$u\frac{\partial N}{\partial x}+v\frac{\partial N}{\partial y}+\frac{b {W}_{c}}{({C}_{w} - {C}_{\infty })}\left[ \frac{\partial }{\partial y}\left(N\frac{\partial C}{\partial y}\right)\right]={D}_{m}\frac{{\partial }^{2}N}{\partial {y}^{2}} .$$

6 $$u\frac{\partial a}{\partial x}+v\frac{\partial a}{\partial y}={D}_{A}\frac{{\partial }^{2}a}{\partial {y}^{2}}-{K}_{c}a{b}^{2},$$

7 $$u\frac{\partial b}{\partial x}+v\frac{\partial b}{\partial y}={D}_{B}\frac{{\partial }^{2}B}{\partial {y}^{2}}+{K}_{c}a{b}^{2},$$

having boundary conditions^67^

8 $$\left.\begin{array}{c}u={U}_{w}=cx, v=0 , - k\frac{\partial T}{\partial y}={h}_{f}\left({T}_{w}-T\right), {D}_{A}\frac{\partial a}{\partial y}={K}_{s}a, \\ {D}_{B}\frac{\partial b}{\partial y}=-{K}_{s}a ,-{D}_{B}\frac{\partial C}{\partial y}={h}_{g}({C}_{w}-C), - {D}_{m}\frac{\partial N}{\partial y}={h}_{n}({N}_{w}-N) at y=0 \\ u\to {U}_{e}=bx, \frac{\partial u}{\partial y}\to 0, T\to {T}_{w},C\to {C}_{w}, N\to {N}_{w}, a\to {a}_{0}, b\to 0 as y\to \infty .\end{array}\right\}$$

The above governing equations are solved by following suitable transformations:

9 $$\left.\begin{array}{l}\Upsilon=y\sqrt{\frac{c}{v}} , u = c x f{^{\prime}} , v = - \sqrt{c v} f , \theta = \frac{T - {T}_{\infty }}{{T}_{w} - {T}_{\infty }} ,\\ \phi = \frac{C - {C}_{\infty }}{{C}_{w} - {C}_{\infty }} , \chi =\frac{N -{N}_{\infty }}{{N}_{w}-{N}_{\infty }},a={a}_{0}h(\eta ), b={b}_{0}q(\eta ).\end{array}\right\}$$

Here f, $\theta$, $\phi ,$ and $\chi$ are functions of $\Upsilon$. Following equations are obtained.

10 $$\left.\begin{array}{l}{f}^{{^{\prime}}{^{\prime}}{^{\prime}}}-M{f}{^{\prime}}-({f}{^{\prime}}{)}^{2}+f{f}^{{^{\prime}}{^{\prime}}}+ \alpha (2{f}{^{\prime}}{f}^{{^{\prime}}{^{\prime}}{^{\prime}}}-f{f}^{iv}-({f}^{{^{\prime}}{^{\prime}}}{)}^{2})-\lambda (\theta -Nr\phi -Nc\chi )\\ +MK+{K}^{2}+\mathrm{H exp}(-\mathrm{\Lambda \Upsilon})=0,\end{array}\right\}$$

11 $$\left.\begin{array}{l}(1+\frac{4}{3} Rd ) \theta {^{\prime}}{^{\prime}} + Pr ( {\delta }_{1} \theta + f \theta {^{\prime}} ) +Pr [- Nb ( f \theta {^{\prime}} \phi {^{\prime}}{^{\prime}} \\ - f \theta {^{\prime}}{^{\prime}} \phi {^{\prime}} )+ 2 Nt f{^{\prime}} \theta {^{\prime}} \theta {^{\prime}}{^{\prime}}-( {f}^{2} \theta {^{\prime}}{^{\prime}} -f f{^{\prime}} \theta {^{\prime}} )]=0\end{array}\right\}$$

12 $$\left.\begin{array}{l}\phi {^{\prime}}{^{\prime}} + Le Pr [ - f \phi {^{\prime}} - {\sigma }^{*} ( 1 + \mu \theta {)}^{n} exp (\frac{-E}{1 + \mu \theta } ) \phi - {\lambda }_{C} {K}_{c} f \phi {^{\prime}} ] \\ + Le Pr {\lambda }_{C}[ - {f}^{2} \phi {^{\prime}}{^{\prime}} - f f{^{\prime}} \phi - Le Pr \frac{Nt}{Nb} ( \theta {^{\prime}}{^{\prime}} + f{^{\prime}} \theta {^{\prime}}{^{\prime}} ) ]=0,\end{array}\right\}$$

13 $${\chi }{{^{\prime}}{^{\prime}}}+Lbf{\chi }{^{\prime}}-Pe\left[(\chi +\omega )\phi {^{\prime}}{^{\prime}}+\chi {^{\prime}}\phi {^{\prime}}\right]=0.$$

14 $$\frac{1}{Sc}{h}^{{^{\prime}}{^{\prime}}}+\frac{2}{3}f{l}{^{\prime}}-Kh{q}^{2}=0,$$

15 $$\frac{{\delta }^{*}}{Sc}{q}^{{^{\prime}}{^{\prime}}}+\frac{2}{3}f{q}{^{\prime}}+Kh{q}^{2}=0,$$

With boundary conditions

16 $$\left.\begin{array}{c}f=0, {f}{^{\prime}}=1, {\theta }{^{\prime}}=-{\gamma }_{1}\left(1-\theta \left(\Upsilon\right)\right), {\phi }{^{\prime}}=-{\gamma }_{2}\left(1-\phi \left(\Upsilon\right)\right),\\ {h}{^{\prime}}(\eta )={K}_{1}h, \delta {q}{^{\prime}}(\eta )=-{K}_{1}h, {\chi }{^{\prime}}=- {\gamma }_{3}\left(1-\chi \left(\Upsilon\right)\right) at \Upsilon=0, \\ {f}{^{\prime}}\to K,{f}^{{^{\prime}}{^{\prime}}}\to 0, \theta \to 0, \phi \to 0, \chi \to 0, h(\eta )\to h, q(\eta )\to 0 as \Upsilon\to \infty .\end{array}\right\}$$

In most cases, the diffusion coefficients ${D}_{A}$=${D}_{B}$ gives $\delta =1$. The following relation can be achieved after the implementation of this assumption.

17 $$h(\eta )+q(\eta )=1,$$

utilizing (17) in (14) and (15) gives

18 $$\frac{1}{Sc}{h}^{{^{\prime}}{^{\prime}}}+\frac{2}{3}f{h}{^{\prime}}-{K}_{1}h(1-h{)}^{2}=0,$$

whereas boundary conditions are bestowed by

19 $${h}{^{\prime}}(0)={K}_{2}h(0), h(\infty )=1.$$

Different dimensionless parameters arising in (9)–(13) are given underneath:

20 $$\left.\begin{array}{ll}& \alpha =\frac{{cK}_{0}}{\mu }, K=\frac{\mathrm{b}}{c}, M=\sqrt{\frac{\sigma {B}_{0}^{2}}{{\rho }_{f}}},\mathrm{ H}=\frac{\pi {j}_{0}{M}_{0}}{8\rho {U}_{w}^{2}},\Lambda =\sqrt{\frac{{\pi }^{2}\nu }{{a}^{2}c}}\\ & \lambda =\frac{(1-{C}_{\infty }){\beta }^{**}{g}^{*}({T}_{w}-{T}_{\infty })}{{c}^{2}x},Nr=\frac{({\rho }_{p}-{\rho }_{f}){g}^{*}({C}_{w}-{C}_{\infty })}{(1-{C}_{\infty }){\beta }^{**}{\rho }_{f}({T}_{w}-{T}_{\infty })},\\ & {\delta }_{1}=\frac{{Q}_{0}}{c\rho {C}_{p}}, Pr=\frac{\nu }{\alpha }, Nc=\frac{({\rho }_{m}-{\rho }_{f})\gamma ({N}_{w}-{N}_{\infty })}{(1-{C}_{\infty }){\beta }^{**}{\rho }_{f}({T}_{w}-{T}_{\infty })},\\ & Nb=\frac{\tau {D}_{B}({C}_{w}-{C}_{\infty })}{\nu }, Rd=\frac{4{\sigma }^{*}{T}_{\infty }^{3}}{{k}^{*}}, Nt=\frac{\tau {D}_{T}({T}_{w}-{T}_{\infty })}{\nu {T}_{\infty }},\\ & {\lambda }_{C}=c{\lambda }_{c}^{*}, Le=\frac{\alpha }{{D}_{B}}, E=\frac{-{E}_{a}}{\kappa {T}_{\infty }}, \mu =\frac{({T}_{w}-{T}_{\infty })}{{T}_{\infty }}, {\sigma }^{*}=\frac{K{r}^{2}}{c},Sc=\frac{\nu }{{D}_{B}},\\ & \frac{Nt}{Nb}=\frac{{D}_{T}({T}_{w}-{T}_{\infty })}{{D}_{B}{T}_{\infty }({C}_{w}-{C}_{\infty })}, Lb=\frac{\nu }{{D}_{m}}, Pe=\frac{b{W}_{c}}{{D}_{m}},{K}_{1}=\frac{{K}_{c}{a}_{0}}{{x}^{\frac{-2}{3}}}, {K}_{2}=\frac{{K}_{s}{a}_{0}^{2}}{l}.\end{array}\right\}$$

where $\alpha$ is a viscoelastic parameter while $\mathrm{K}$ is a velocity ratio parameter. $M$,$H,\Lambda$, $Nr,$ and $Nc$ are signifying magnetic field parameter, modified Hartman number, dimensionless parameter, Buoyancy ratio number, and rayleigh number of bioconvection, respectively. $Pr$ is the Prandtl number, while $Nb$ is utilized as a parameter for Brownian motion. ${\delta }_{1}$ is used for a different number of microorganisms, whereas $Rd$ is a parameter for thermal radiation. $Le$ and $E$ are showing as Lewis number and activation energy. $\mu$ is the temperature difference parameter while ${\sigma }^{*}$ is a chemical reaction factor. $Lb$ is the Lewis parameter of bio-convection, and $Pe$ is used as the Peclet parameter. Also, ${\gamma }_{1}$, ${\gamma }_{2}$, ${\gamma }_{3}$, $Nt$ and $\lambda$ are signifying Biot number of heat, Biot number of solution, Biot number of microorganisms, Thermophoresis parameter, and parameter of mixed convection, ${K}_{1}$ and ${K}_{2}$ denotes the parameters of homogeneous and heterogeneous reactions, respectively.

## Galerkin-finite element method (G-FEM)

**Figure 2:**
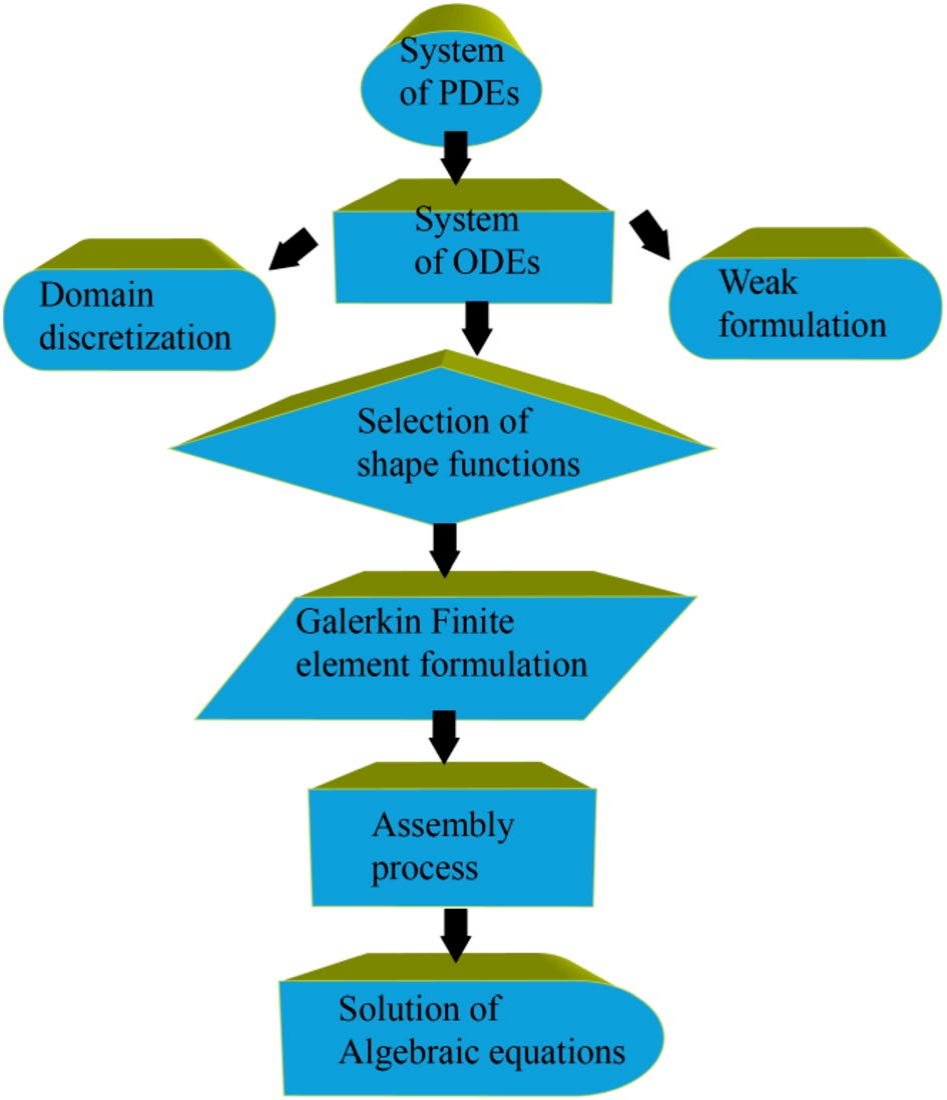
Flow chart of G-FEM.

The corresponding boundary constraints of the present system were computational simulated using FEM. FEM is based on partitioning the desired region into components (finite). FEM^68^ is covered in this section. Figure 2 depicts the flow chart of the finite element method. This method has been employed in numerous computational fluid dynamics (CFD) issues; the benefits of employing this strategy are discussed further below.

Weak form is derived from strong form (stated ODEs), and residuals are computed. To achieve a weak form, shape functions are taken linearly, and FEM is used. The assembly method builds stiffness components, creating a global stiffness matrix. An algebraic framework (nonlinear equations) is produced using the Picard linearizing technique. Algebraic equations are simulated utilizing appropriate halting criterion through 10^–5^ (supercomputing tolerances).

$$\left|\frac{{\Upsilon}_{i+1}-{\Upsilon}_{i}}{{\Upsilon}^{i}}\right|<{10}^{-5}.$$

Further, The Galerkin finite element technique's flow chart is depicted in Fig. 2.

## Validity of code

The validity of the computational technique was determined by comparing the heat transmission rate from the existing method to the verified consequences of earlier investigations^69–71^. A comparison of current research outcomes with the previous study results is shown in Table 1. The current study is comparable and presented highly accurate results.

**Table 1:** Comparing ${-\theta }{^{\prime}}\left(0\right)$ with alteration in Prandtl number, and taking ${\delta }_{1}=Rd=Nt={\gamma }_{1} ={\gamma }_{2}= {\gamma }_{3}=0$ and $Nb=0$.

${P}_{r}$	Das et al.^69^	Qureshi^70^	Hussian^71^	This study
0.72	0.80876122	0.8087618	0.8087618	0.8087612
1.0	1.00000000	1.0000000	1.0000000	1.0000000
3.0	1.92357431	1.9235742	1.9235742	1.9235734
7.0	3.07314679	3.0731465	3.0731465	3.0731465
10	3.72055436	3.7205542	3.7205542	3.7205511

The important physical parameters in this problem are the local heat and mass fluxes, as well as the local motile microorganisms flux, which are specified as:

21 $$N{u}_{x}=\frac{x{q}_{w}}{k({T}_{w}-{T}_{\infty })}, S{h}_{x}=\frac{x{q}_{m}}{{D}_{B}({\phi }_{w}-{\phi }_{\infty })},N{n}_{x}=\frac{x{q}_{n}}{{D}_{m}({N}_{w}-{N}_{\infty })}$$

in which

22 $${q}_{w}=-k{\left(\frac{\partial T}{\partial y}\right)}_{y=0},{q}_{m}=-{D}_{B}{\left(\frac{\partial \phi }{\partial y}\right)}_{y=0},{q}_{n}=-{D}_{m}{\left(\frac{\partial N}{\partial y}\right)}_{y=0 }.$$

Using the similarity transform presented above, Eq. (23) can be described as:

23 $$N{u}_{x}R{e}_{x}^{-1/2} = -{\theta }{^{\prime}}(0),S{h}_{x}R{e}_{x}^{-1/2} = -{\phi }{^{\prime}}(0), N{n}_{x}R{e}_{x}^{-1/2}=-{\chi }{^{\prime}}(0)$$

## Results and discussion

This portion of the analysis demonstrates the effects of the pertinent parameters on the dimensionless boundary layer distributions.

**Figure 3:**
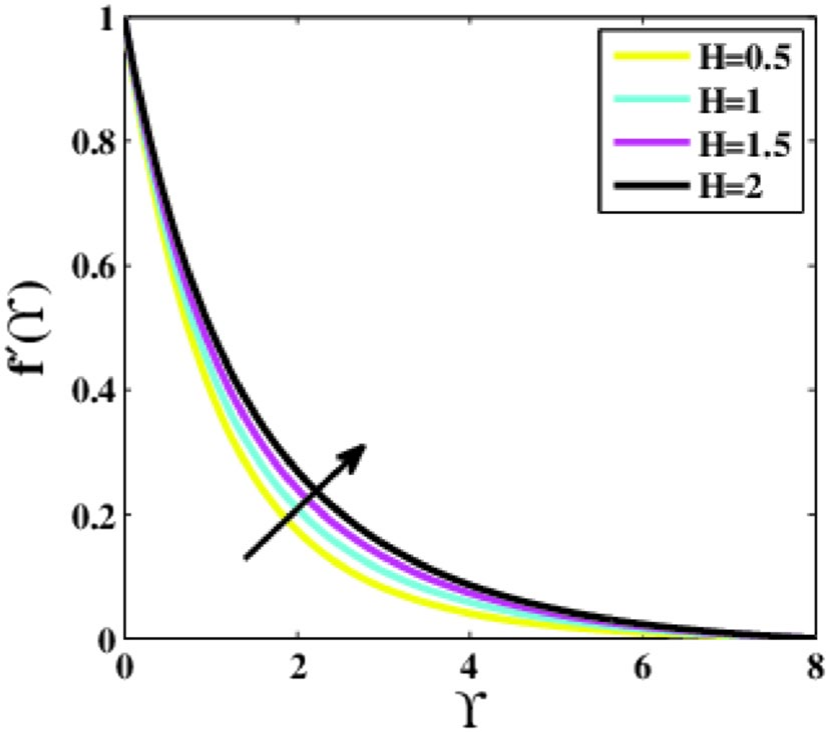
Plots of ${f}{^{\prime}}(\Upsilon )$ for $H$.

**Figure 4:**
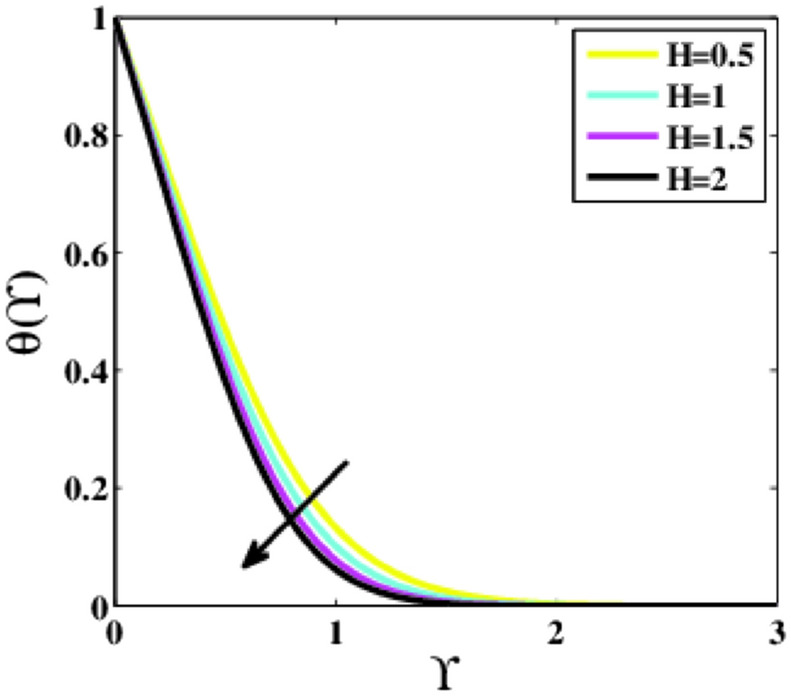
Plots of $\theta (\Upsilon )$ for $H$.

Figures 3, 4 are depicted to analyze the effects of the modified Heart number $H$ on the velocity $f{^{\prime}}\left(\Upsilon\right)$ and temperature $\theta \left(\Upsilon\right)$. It is worth illustrating that the profile $f{^{\prime}}\left(\Upsilon\right)$ is enhancing for enhancing values of the parameter $H,$ whereas the profile $\theta \left(\Upsilon\right)$ decays. An acceleration is noticed in the velocity as the strength of the parameter $H$ is escalated. This makes good sense given the physics of the problem,$H>0$ aids the flow phenomena during flow dispersion. This trend demonstrates that upon escalating the strength of the parameter $H,$ the outward electric field becomes stronger. The velocity profile grew as a result. In these conditions, the magnetic field’s strength and electromagnetic field increase equivalently. Additionally, the Riga plate induced Lorentz power that corresponded to the surface and caused additional surface strain, leading to a rise in the fluid’s speed.

**Figure 5:**
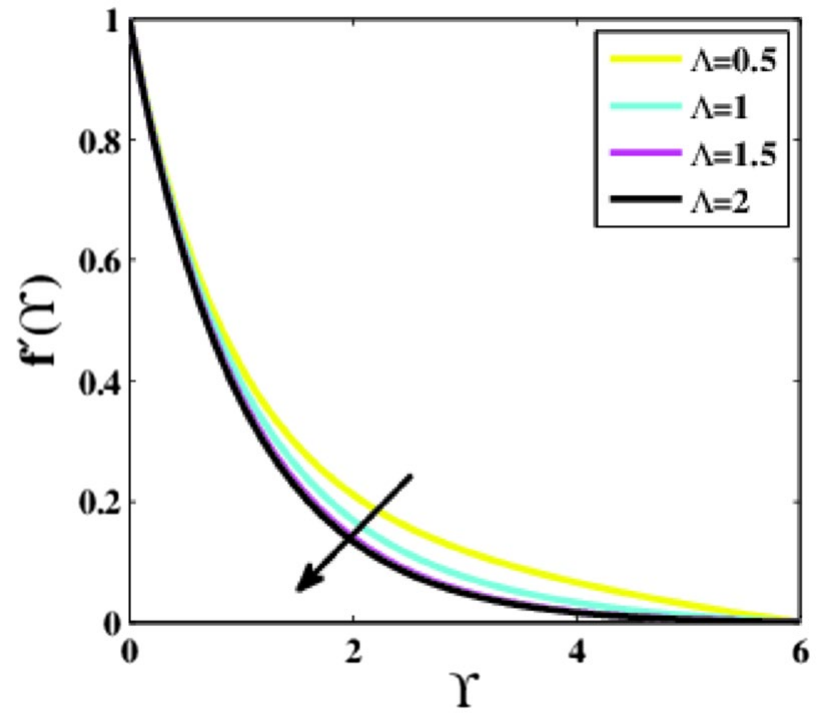
Plots of ${f}{^{\prime}}(\Upsilon )$ for $\Lambda$.

The impact of the dimensional parameter $\Lambda$ on the velocity profile $f{^{\prime}}\left(\Upsilon\right)$ is illustrated in Fig. 5. It is experienced that the profile $f{^{\prime}}\left(\Upsilon\right)$ is decaying for enhancing values of the parameter $\Lambda$. It is because the thickness of the boundary layer deteriorates as the parameter $\Lambda$ improves, so the velocity reduces within the boundary layer.

**Figure 6:**
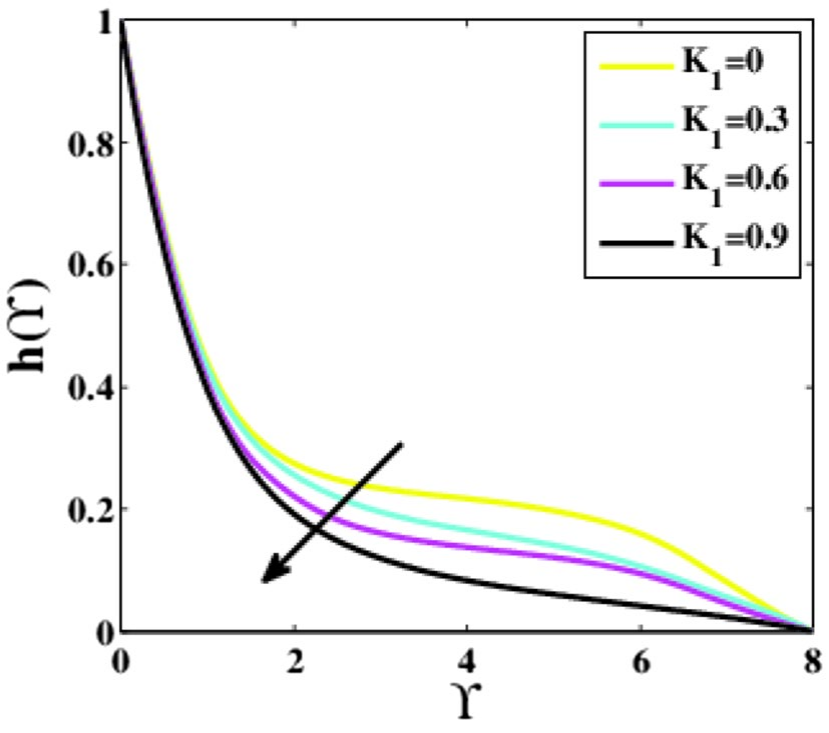
Plots of $h(\Upsilon )$ for ${K}_{1}.$

**Figure 7:**
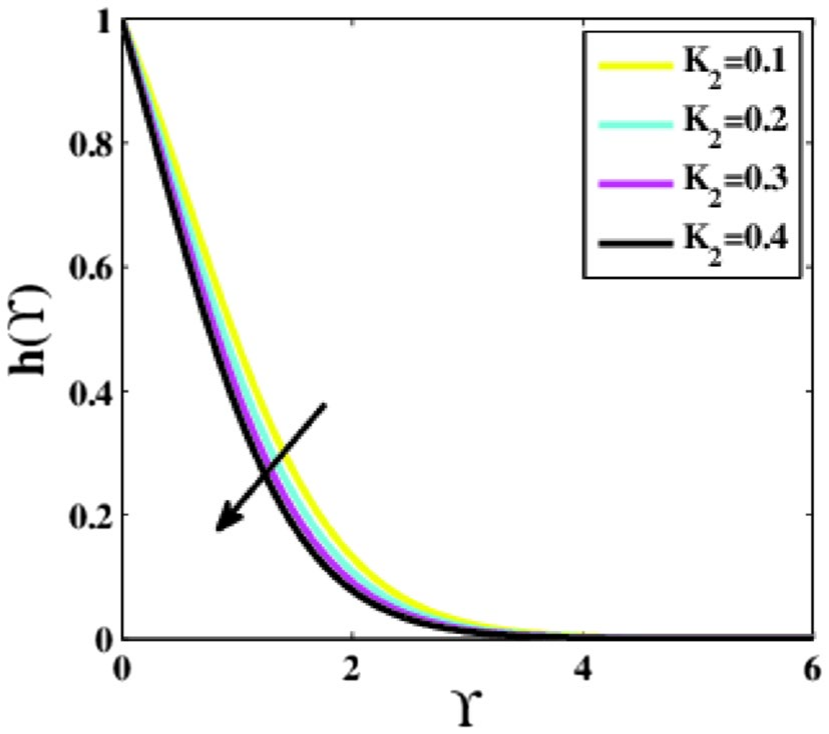
Plots of $h(\Upsilon )$ for ${K}_{2}$.

The impacts of the homogeneous and heterogeneous chemical parameters $\left({K}_{1}, {K}_{2}\right)$ on the profile $h\left(\Upsilon\right)$ are captured in Figs. 6, 7. It is examined that the profile $h\left(\Upsilon\right)$ reduces for higher estimations of both parameters. With escalated values of the parameters ${K}_{1}, {K}_{2}$ the reactants are consumed, which is responsible for the reduction in the profile $h\left(\Upsilon\right)$ and boundary layer.

**Figure 8:**
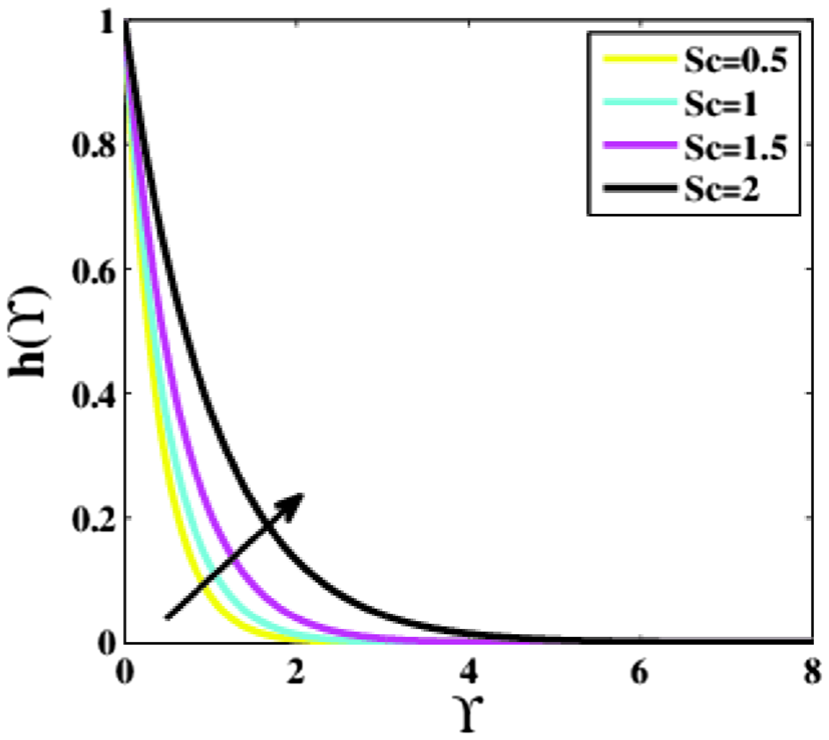
Plots of $h(\Upsilon )$ for $Sc$.

Also, Fig. 8 portrays that upon mounting the Schmidt number $Sc,$ the profile $h\left(\Upsilon\right)$ grows. Since the Schmidt number is defined as the ratio of momentum to mass diffusivity. Therefore, the parameter $Sc$ is increased by large momentum diffusivity relative to mass diffusivity. It finally results in a higher profile $h\left(\Upsilon\right)$.

**Figure 9:**
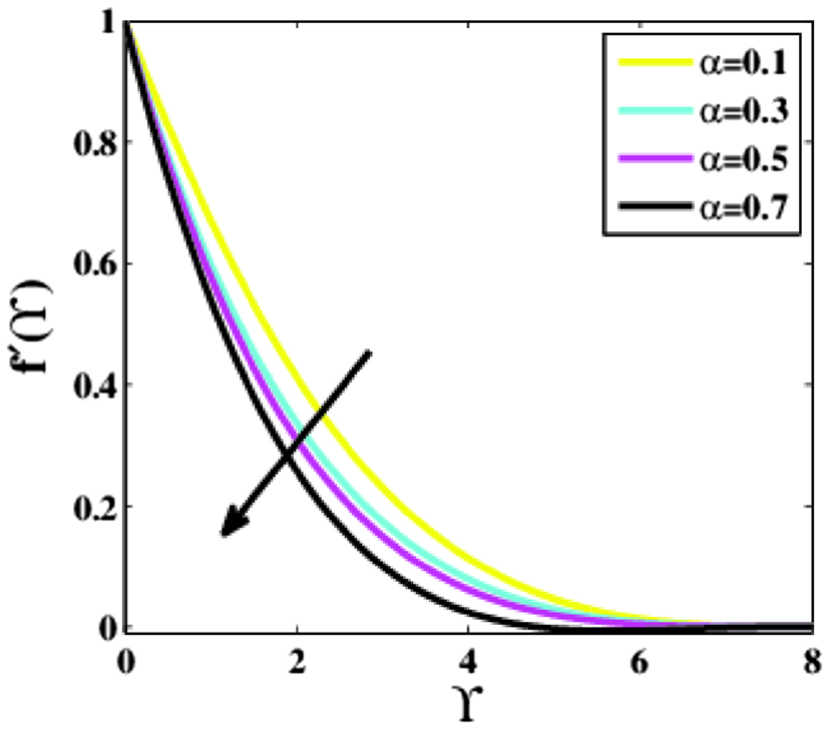
Plots of ${f}{^{\prime}}(\Upsilon )$ for $\alpha .$

Figure 9 shows that the velocity profile is observed decaying for the various alterations in the viscoelastic parameter. As the parameter $\alpha$ surges, a noticeable decrement in profile $f{^{\prime}}\left(\Upsilon\right)$ is seen. It is because the tensile stress produced by the viscoelastic parameter causes the thickness of the boundary layer to drop and compares transversely, lowering the velocity.

**Figure 10:**
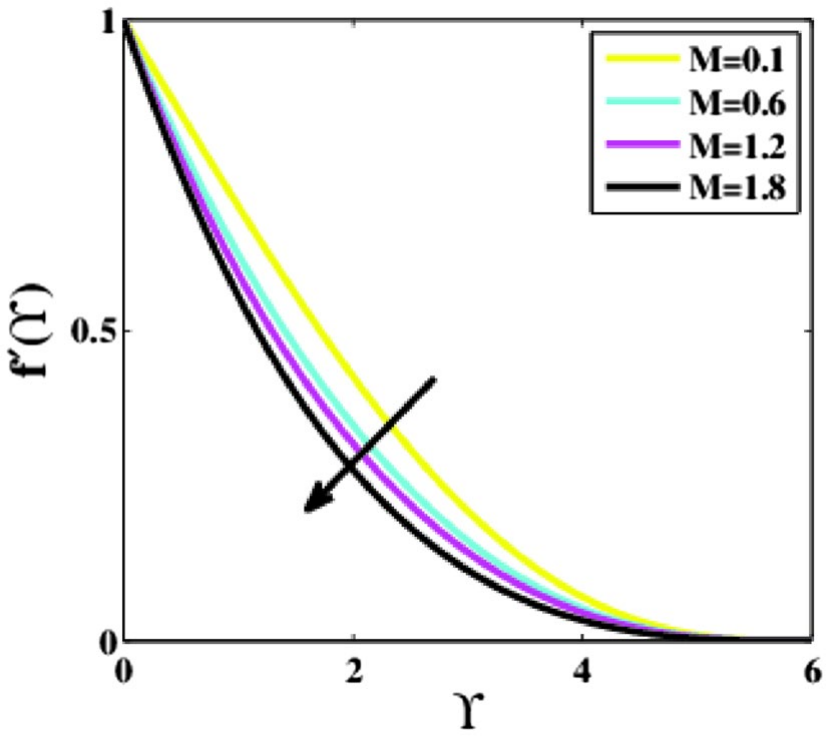
Plots of ${f}{^{\prime}}(\Upsilon )$ for $M$.

Figure 10 portrays the impression of the magnetic parameter $M$ on the dimensionless velocity profile $f{^{\prime}}\left(\Upsilon\right)$ along $\Upsilon$. With rising values of the parameter $M,$ the flow velocity profile is observed to decrease noticeably over the fluid domain. When a magnetic field is applied to a fluid that is electrically conducting, it creates a drag-like force known as Lorentizian force. This force produces friction in the direction of the fluid, and as a result, the fluid velocity decays. The velocity is maximum when $M=0.1$, but when $M$ rises, the velocity profile decreases.

**Figure 11:**
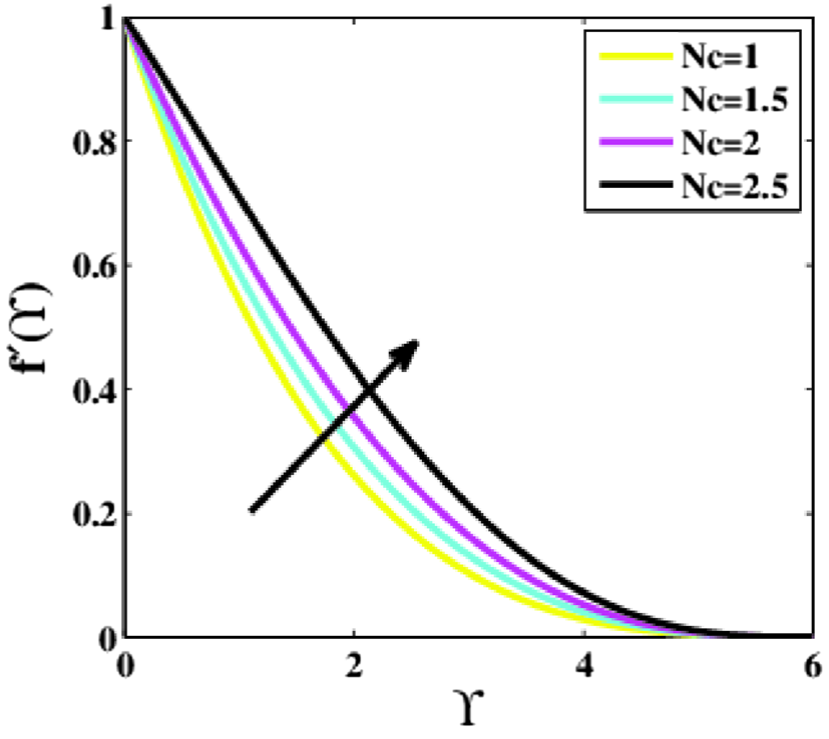
Plots of ${f}{^{\prime}}(\Upsilon )$ for $Nc$.

Figure 11 is drawn to analyze the influence of the Rayleigh number $Nc$ on the non-dimensional velocity profile $f{^{\prime}}\left(\Upsilon\right)$. A noticeable increment in the velocity profile is seen for higher estimations of the parameter $Nc$. For $Nc=1.0$ the velocity has obtained its maximum value than from the other estimations of $Nc$.

**Figure 12:**
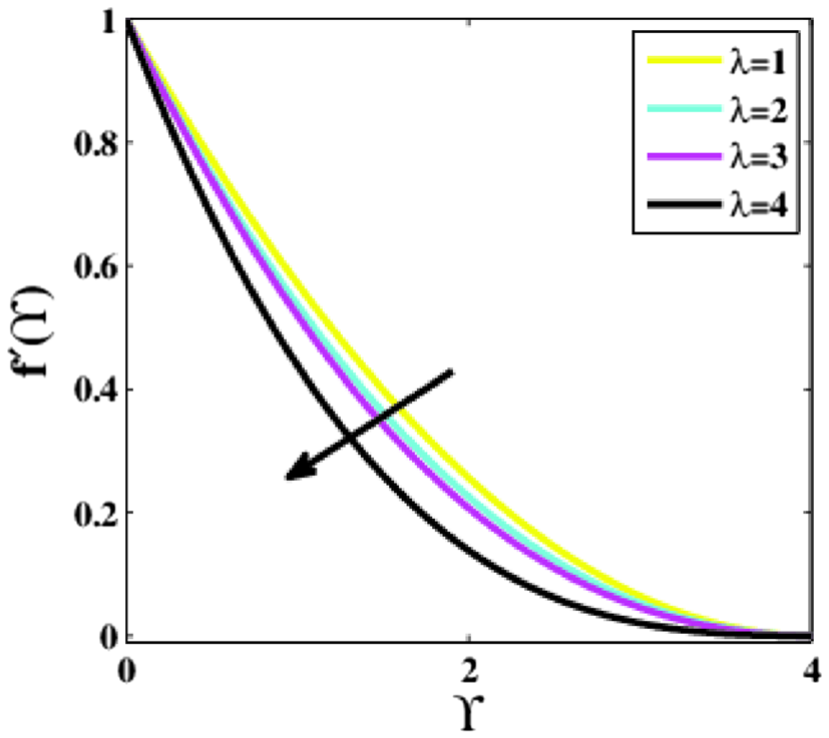
Plots of ${f}{^{\prime}}(\Upsilon )$ for $\lambda$.

Figure 12 measures the effectiveness of the mixed convection parameter $\lambda$ on the dimensionless velocity profile $f{^{\prime}}\left(\Upsilon\right)$ along the dimensionless similarity variable $\Upsilon$. A considerable decrement in the profile is seen. A large difference is observed in the decrement of the fluid velocity profile for $\lambda =4$ from $\lambda =1.0, 2.0, \mathrm{and}\,\, 3.0$.

**Figure 13:**
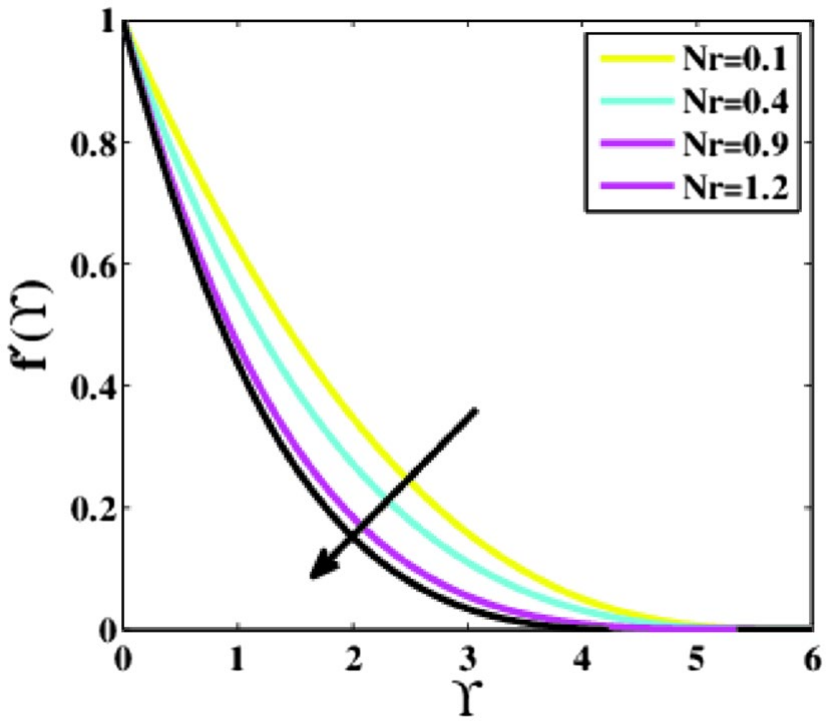
Plots of ${f}{^{\prime}}(\Upsilon )$ for $Nr$.

Figure 13 predicts the influence of the Buoyancy ratio parameter $Nr$ on the non-dimensional profile of the velocity $f{^{\prime}}\left(\Upsilon\right)$ alongside $\Upsilon$. The profile $f{^{\prime}}\left(\Upsilon\right)$ decreases for larger $Nr$. The occurrence of nanoparticles increases the opposing buoyancy, causing the fluid velocity to diminish. Also, the effects for $Nr=1.2$ are smaller as compared to $Nr=0.1, 0.4,$ and $0.9$.

**Figure 14:**
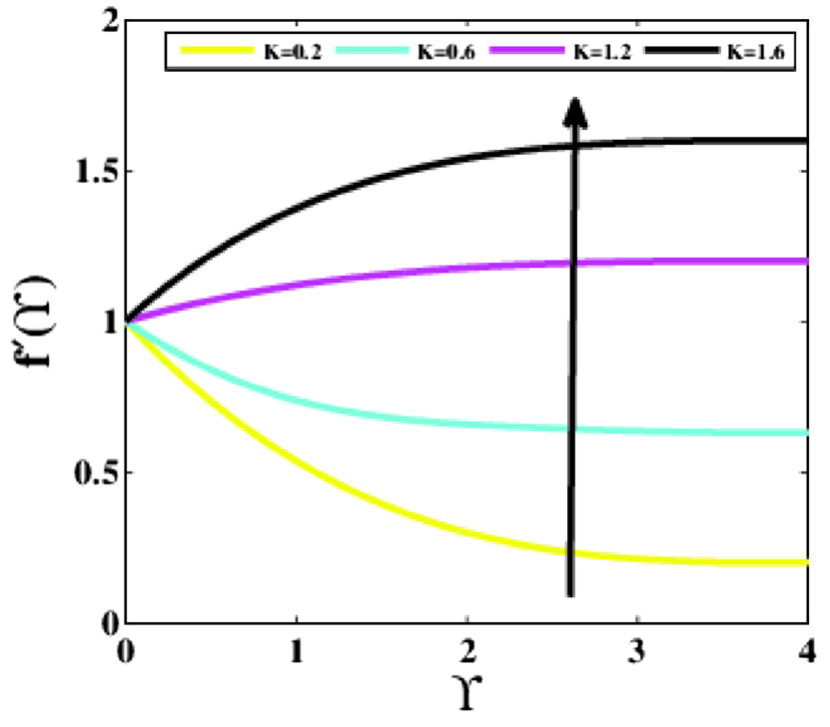
Plots of ${f}{^{\prime}}(\Upsilon )$ for $K$.

Figure 14 signifies the impression of the velocity ratio parameter $K$ on the non-dimensional velocity distribution $f{^{\prime}}\left(\Upsilon\right)$. An immense fluctuation in the fluid velocity and the corresponding thickness of the boundary layer is experienced at the points where free stream velocity differs from the Riga plate’s velocity. As the velocity ratio parameter increases, the velocity rises, and the thickness of the boundary layer falls.

**Figure 15:**
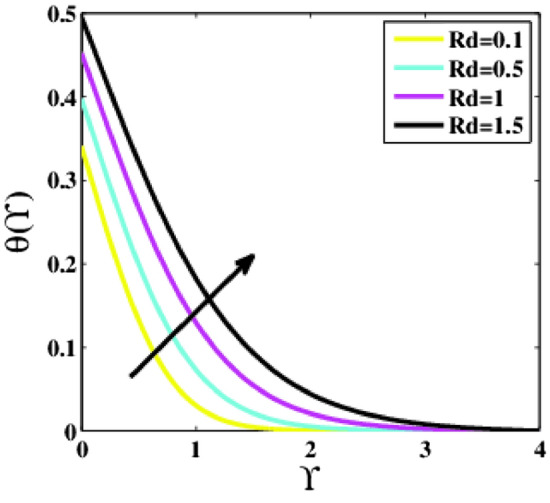
Plots of $\theta (\Upsilon )$ for $Rd$.

Figure 15 depicts the temperature profile $\theta \left(\Upsilon\right)$ for various values of the radiation parameter $Rd$. A reasonable increment in temperature profile is observed for growing values of the parameter $Rd$. The radiation parameter $Rd$ specifies how much conduction heat transfer contributes to thermal radiation transfer. Thus it is self-evident that raising the parameter $Rd$ rises the temperature inside the boundary layer. Because escalation in $Rd$ means a reduction in the co-efficient of mean absorption, which delivers additional heat towards flow, and as a result, an increase in the temperature profile is noticed.

**Figure 16:**
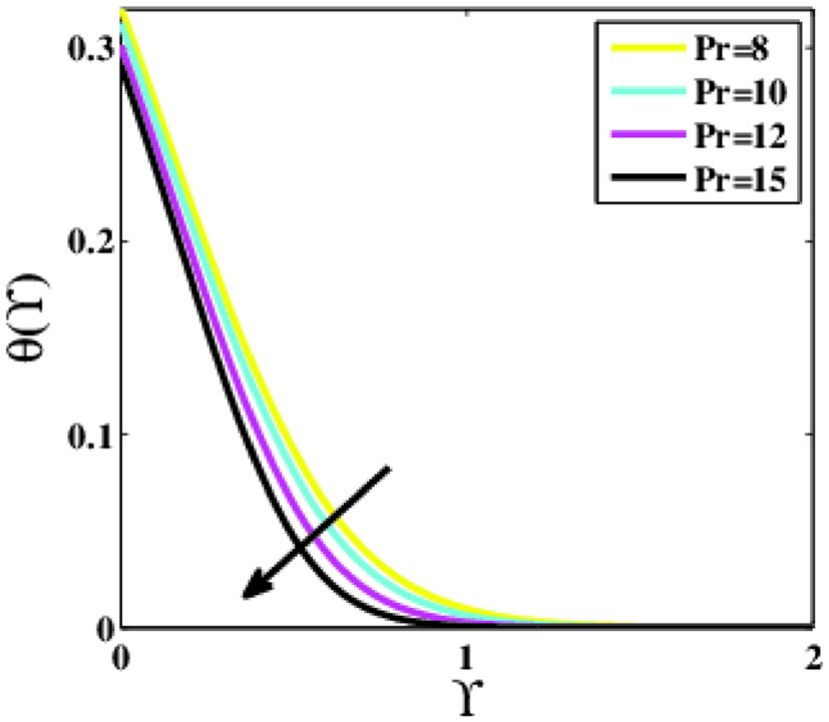
Plots of $\theta (\Upsilon )$ for $Pr$.

Figure 16 depicts the impact of the Prandtl number $Pr$ on the non-dimensional temperature profile $\theta \left(\Upsilon\right)$. The numerical values of the parameter $Pr$ reveal that rising the Prandtl number influences lowering the temperature. A rise in the Prandtl number causes the thickness of the thermal boundary layer to decrease and the average temperature inside the boundary layer to fall. The idea for this is that lower $Pr$ values equate to greater thermal conductivities, allowing heat to escape away from the warmed surface more quickly than with larger $Pr$ values. As a result, the boundary layer becomes thicker for fewer Prandtl numbers, and the heat transfer rate decreases.

**Figure 17:**
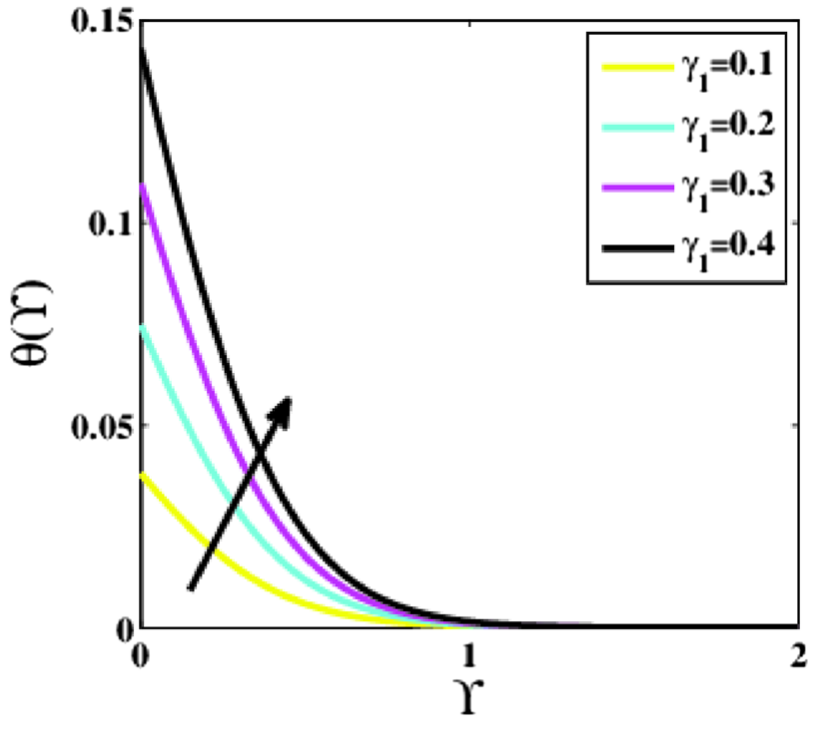
Plots of $\theta (\Upsilon )$ for ${\gamma }_{1}$.

Figure 17 aims to present the impact of the variation in the Biot number of heat ${\gamma }_{1}$ on the non-dimensional temperature profile $\theta \left(\Upsilon\right)$. The profile is witnessed escalating for growing values of the Biot number. Because the Biot number and coefficient of heat transmission are inextricably related. As the parameter ${\gamma }_{1}$ grows, the fluid conductivity decays, but the heat transmission coefficient rises, raising the fluid’s temperature.

**Figure 18:**
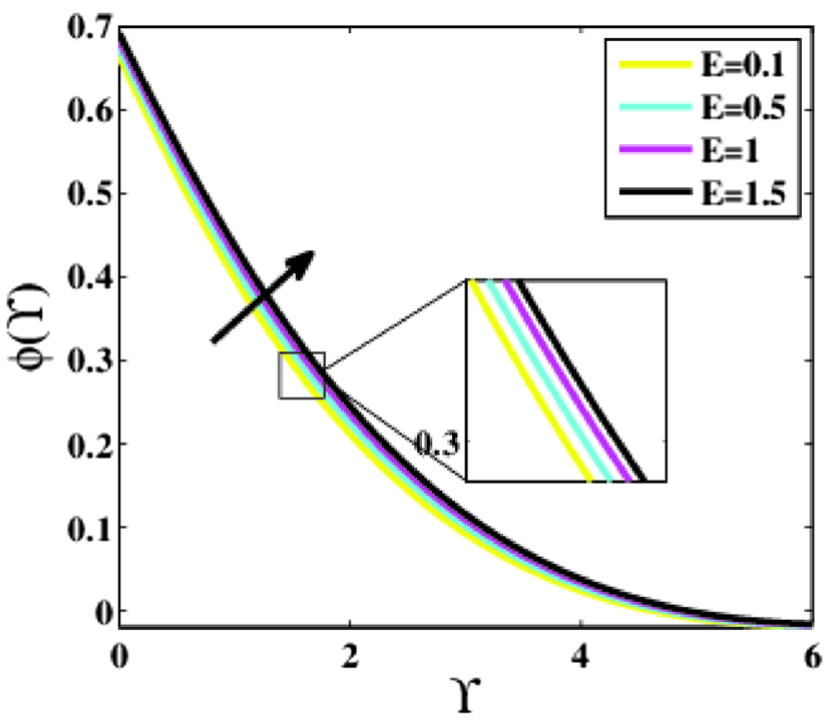
Plots of $\phi (\Upsilon )$ for $E$.

Figure 18 demonstrates the effects of the activation energy parameter $E$ on the concentration profile $\phi \left(\zeta \right)$. The rise in the parameter $E$ leads to an increase in the profile $\phi \left(\Upsilon\right)$. The deterioration of the modified Arrhenius function is caused by increasing $E$. Eventually, this encourages productive chemical processes, resulting in increased concentration. The term $\phi exp\left(\frac{-E}{1+\mu \theta }\right)$ in the Eq. (11) is a more useful tool for determining how Arrhenius activation influences nanoparticle concentration.

**Figure 19:**
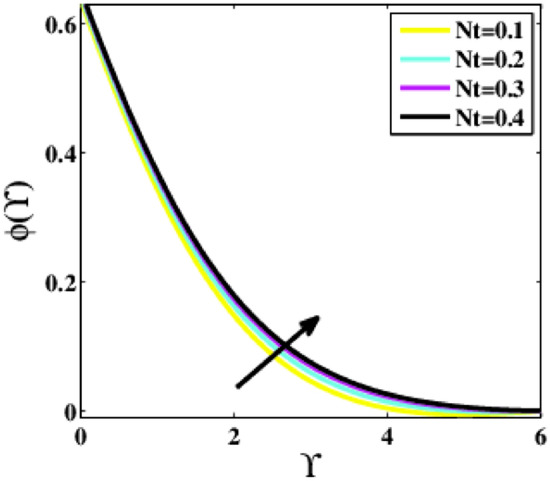
Plots of $\phi (\Upsilon )$ for $Nt$.

**Figure 20:**
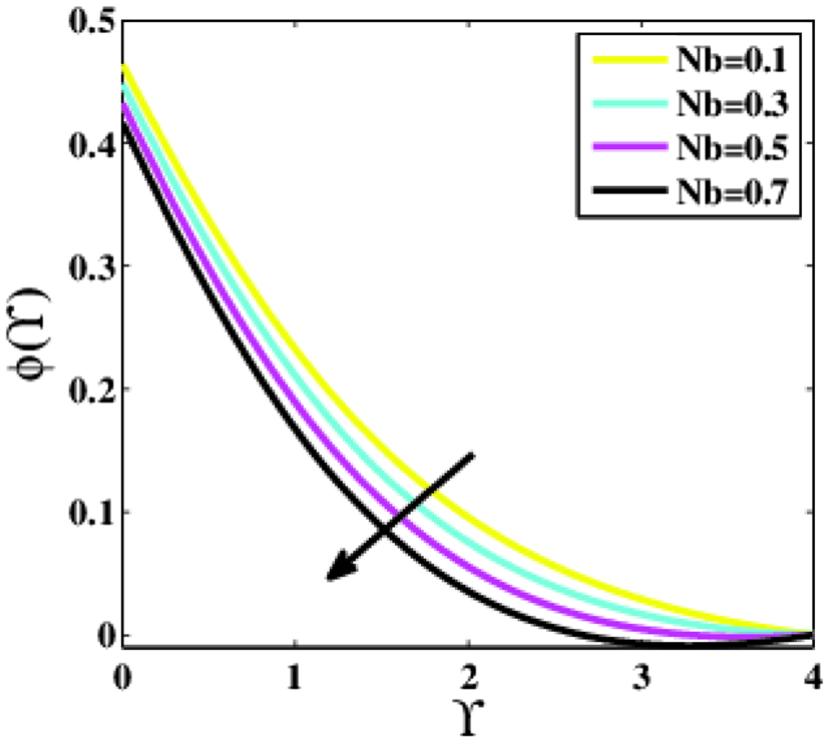
Plots of $\phi (\Upsilon )$ for $Nb$.

Figure 19, 20 are extracted to predict the effects of the thermophoresis and Brownian motion parameters $\left(Nt ,Nb\right)$. For varying values of the parameters $Nt$ and $Nb$, the profile $\phi \left(\Upsilon\right)$ explores two possible patterns. As the parameter $Nt$ rises, the profile $\phi \left(\Upsilon\right)$ in Fig. 19 rises as well because nanoparticles are distributed from the hot to the ambient fluid during the thermophoresis process, and as a result, nanoparticle concentration augments. Whereas the concentration in Fig. 20 diminishes as the Brownian motion parameter rises. Th Brownian motion is created by nanoparticles colliding with a base fluid. The arbitrary motion of nanoparticles increases the kinetic energy unless the Brownian motion has a major influence on nanoparticle diffusion.

**Figure 21:**
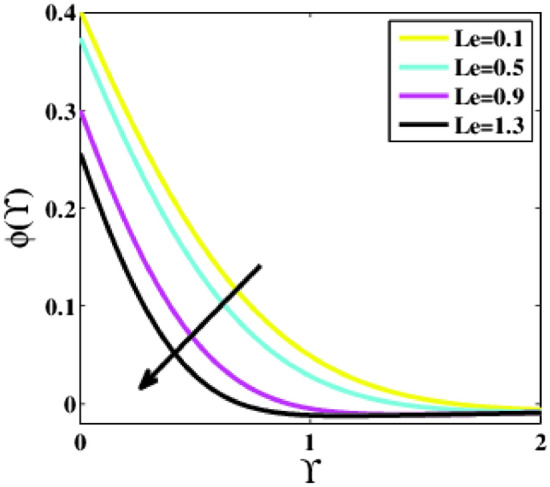
Plots of $\phi (\Upsilon )$ for $Le$.

Figure 21 interprets the impact of the Lewis number $Le$ on the non-dimensional concentration profile $\phi \left(\Upsilon\right)$*.* Clearly, the profile $\phi \left(\Upsilon\right)$ reduces as the parameter $Le$ enhances. Lewis number denotes the relative contribution of the thermal to the species diffusion rate within the boundary layer region. According to the definition, the parameter $Le$ is basically the ratio of Schmidt to Prandtl number. As a result, when $Le=1.0,$ heat and species disperse at equal speed. When $Le>1.0$, heat diffuses more quickly than species. The boundary layer of species nanoparticle concentration grows steeper when the Lewis number is enhanced.

**Figure 22:**
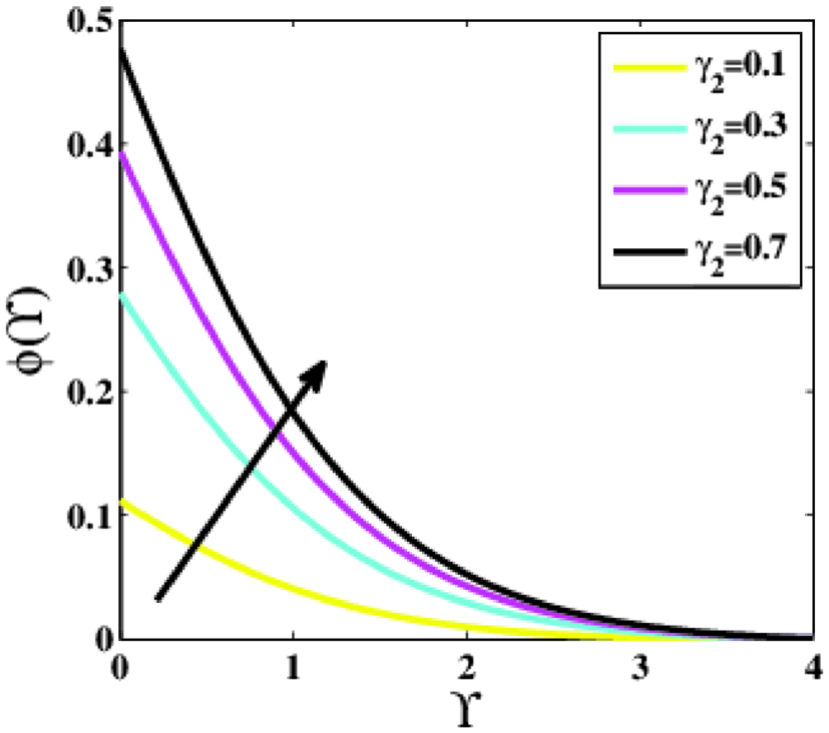
Plots of $\phi (\Upsilon )$ for ${\gamma }_{2}$.

Figure 22 elucidates the impression of the Biot number of solution ${\gamma }_{2}$ on the dimensionless concentration profile $\phi \left(\Upsilon\right)$. It has been observed that as the parameter ${\gamma }_{2}$ increases the profile $\phi \left(\Upsilon\right)$ increases. According to the definition of the Biot number, the transferred mass will be spread throughout the surface by convection. As a result, the profile of nanoparticle concentration is amplified.

**Figure 23:**
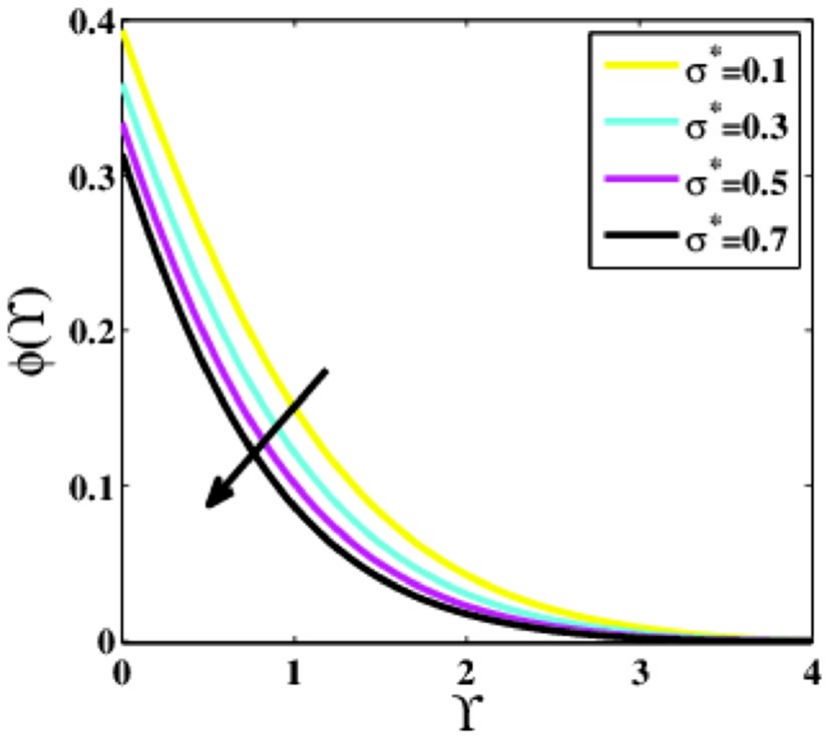
Plots of $\phi (\Upsilon )$ for ${\sigma }^{*}$.

Figure 23 captures the impression of the chemical reaction factor ${\sigma }^{*}$ on the concentration profile $\phi \left(\Upsilon\right)$. The profile $\phi \left(\Upsilon\right)$ is decreasing for growing values of the parameter ${\sigma }^{*}$. When the parameter ${\sigma }^{*}$ increases, the number of solute molecules involved in the reaction increases, lowering the concentration. As a result of the destructive chemical reaction, the solutal boundary layer’s thickness is considerably reduced.

**Figure 24:**
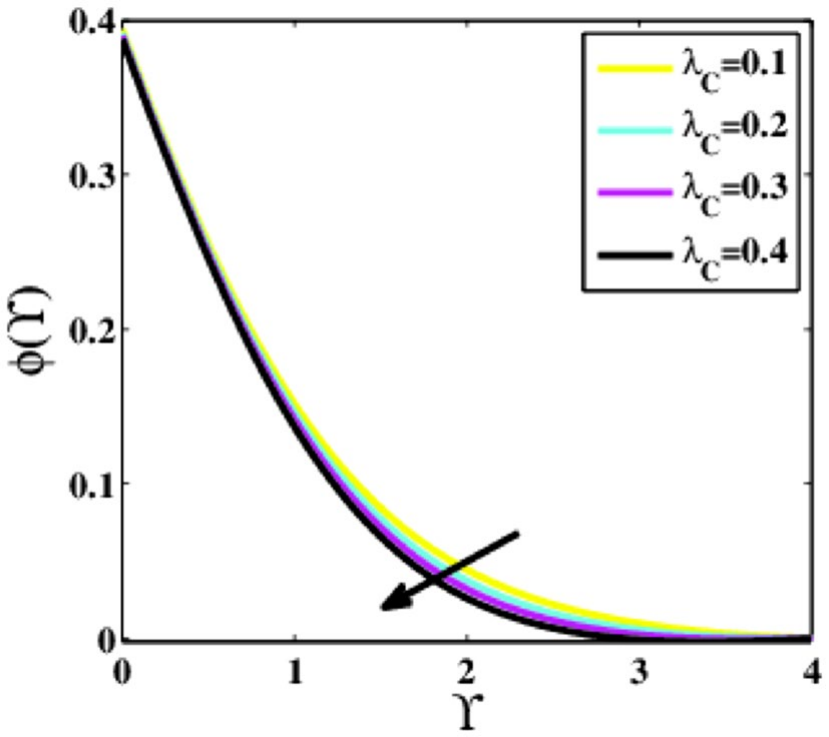
Plots of $\phi (\Upsilon )$ for ${\lambda }_{C}$.

Figure 24 indicates the effects of the relaxation time of the mass parameter ${\lambda }_{C}$ upon the dimensionless concentration profile $\phi \left(\Upsilon\right)$. The profile $\phi \left(\Upsilon\right)$ reduces as the parameter ${\lambda }_{C}$ surges. It is because if the parameter ${\lambda }_{C}$ is larger, then the particles of the fluid will take longer to disperse, resulting in a decrease in the concentration profile.

**Figure 25:**
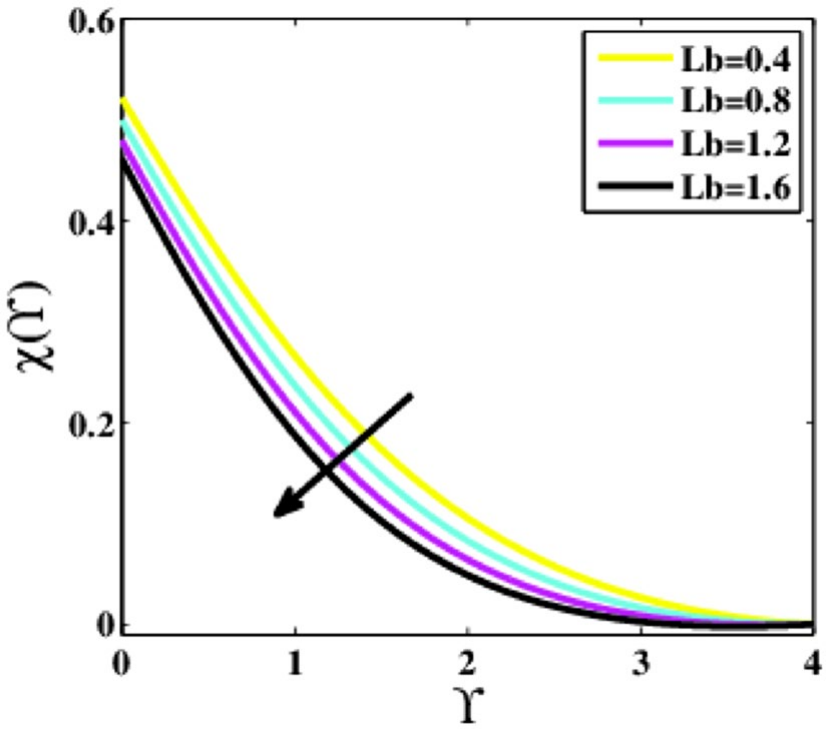
Plots of $\chi (\Upsilon )$ for $Lb$.

Figure 25 is plotted to predict the effects of the bio-convection Lewis number $Lb$ upon the dimensionless profile of microorganisms $\chi \left(\Upsilon\right)$. It is noticed that the profile $\chi \left(\Upsilon\right)$ observe to decrease for higher $Lb$. Physically, the parameter $Lb$ is inversely proportional to the thermal diffusivity; raising $Lb$ lessens the thermal diffusivity, resulting in a decrease in the motile density of microorganisms.

**Figure 26:**
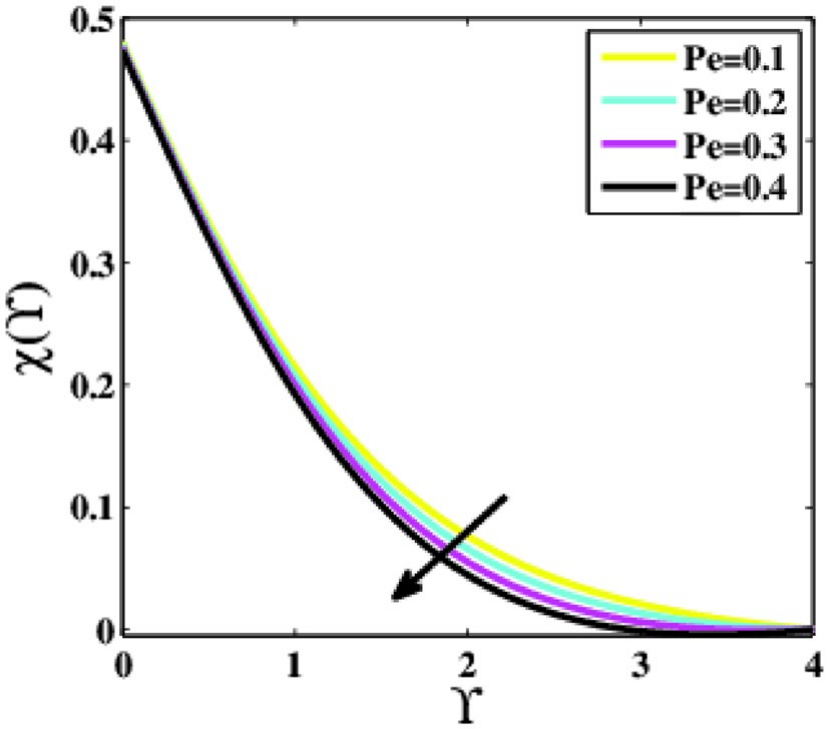
Plots of $\chi (\Upsilon )$ for $Pe$.

Figure 26 is delineated to determine the effects of the Peclet number $Pe$ on the non-dimensional microorganisms profile $\chi \left(\Upsilon\right)$. It is perceived that the profile $\chi \left(\Upsilon\right)$ is decaying for greater estimations of the parameter $Pe$. Physically, the parameter $Pe$ is inversely linked with the diffusivity of the microorganisms and has a direct association with cell swimming speed. Consequently, an increase in $Pe$ increases cell swimming speed while reducing microbe diffusivity. As a result, the microorganism’s ability to develop $Pe$ is reduced.

**Figure 27:**
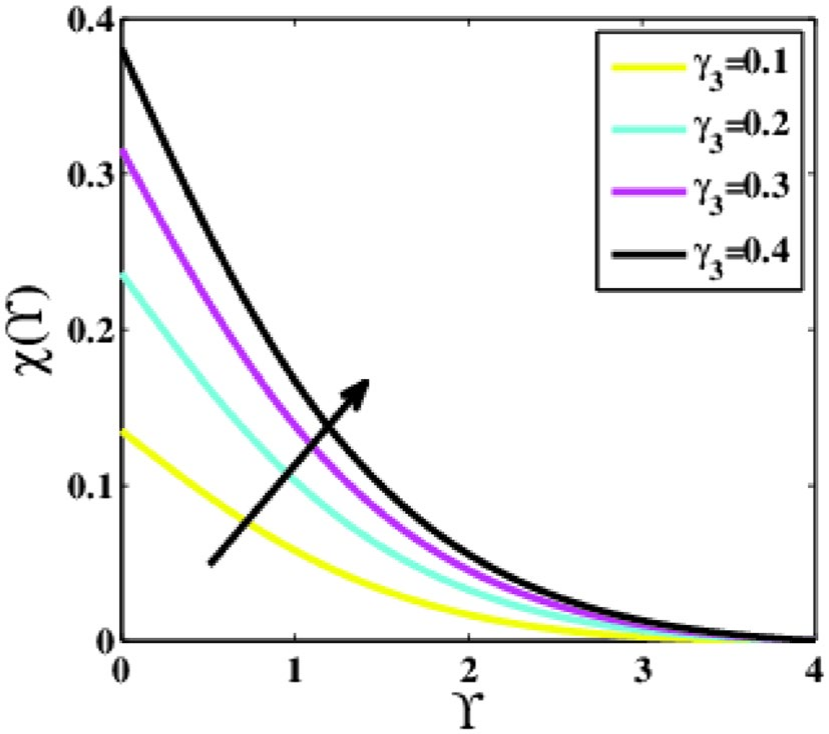
Plots of $\chi (\Upsilon )$ for ${\gamma }_{3}$.

Figure 27 demonstrates the impression of the Biot number of microorganisms ${\gamma }_{3}$ on the non-dimensional profile of the microorganisms $\chi \left(\Upsilon\right)$. The profile $\chi \left(\Upsilon\right)$ escalates for rising values of the parameter ${\gamma }_{3}$. A dramatic increase from ${\gamma }_{3}=0.1$ to ${\gamma }_{3}=0.2$ is noticed.

## Table discussion

The physically interesting quantities like Nusselt number ${N}_{u}R{e}_{x}^{\frac{-1}{2}}$, Sherwood number ${Sh}_{x}R{e}_{x}^{\frac{-1}{2}}$, and local density number ${Nn}_{x}R{e}_{x}^{\frac{-1}{2}}$ are numerically calculated in Table 2 for diverse ranges of the dimensionless parameters like $\mathrm{\alpha }, \lambda , Rd, E, K, Nt, Nb, Lb, H,\Lambda$ and $Le$.

**Table 2:** Nusselt, Sherwood quantities and consistency amount at the stretching walls.

$\lambda$	$\mathrm{\alpha }$	$E$	$Rd$	$K$	$Nb$	$Nt$	$Lb$	$H$	$\Lambda$	Le	${N}_{u}R{e}_{x}^{\frac{-1}{2}}$	${Sh}_{x}R{e}_{x}^{\frac{-1}{2}}$	${Nn}_{x}R{e}_{x}^{\frac{-1}{2}}$
1	1	0.1	0.1	1.1	0.3	0.1	0.4	0.2	0.2	0.5	0.35441	0.29157	0.27379
2											0.33520	0.24121	0.24363
3											0.31624	0.23833	0.22364
	1.0										0.35441	0.29157	0.27379
	0.5										0.31935	0.27364	0.23027
	0.7										0.27162	0.24851	0.21343
		0.1									0.35441	0.29157	0.27379
		0.5									0.38462	0.26493	0.23736
		1									0.40249	0.24436	0.20804
			0.1								0.35441	0.29157	0.27379
			0.5								0.31827	0.32611	0.27379
			1.0								0.28726	0.35923	0.27379
				1.1							0.35441	0.29157	0.27379
				1.2							0.33319	0.31169	0.29849
				1.3							0.30258	0.33113	0.31064
					0.1						0.35441	0.29157	0.27379
					0.3						0.30582	0.34165	0.30862
					0.5						0.25780	0.38861	0.34874
						0.1					0.40663	0.34529	0.31724
						0.2					0.37331	0.32026	0.29136
						0.4					0.35441	0.29157	0.27379
							0.4				0.35441	0.29157	0.27379
							0.8				0.35441	0.29157	0.30412
							1.2				0.35441	0.29157	0.34960
								0.2			0.35441	0.29157	0.27379
								1			0.37545	0.31945	0.30772
								1.5			0.40198	0.33602	0.32159
									0.2		0.35441	0.29157	0.27379
									1		0.38299	0.32169	0.29892
									1.5		0.42102	0.34472	0.31869
										0.1	0.30223	0.27854	0.23857
										0.5	0.35441	0.29157	0.27379
										0.9	0.39964	0.35359	0.29265

## Conclusion

A theoretical analysis has been carried out to describes the characteristics of bioconvection and gyrotactic microorganisms in the MHD flow of Walters-B fluid with homogenous and heterogeneous chemical reactions over a Riga plate. The Buongiorno nanofluid model has been used to model the flow dynamics in terms of highly nonlinear PDEs, which are later transmuted into mixed ODEs with suitable similarity variables. The resultant ODEs are tackled through the assistance of the efficient Galerkin finite element method in COMSOL software. Thus following concluding remarks are drawn from the present analysis:

- The velocity increases for the parameters $Nc \mathrm {and} K$ but is effectively controlled for the parameters $H,\Lambda , \alpha , M, \lambda ,$ and $Nr$.
- The temperature escalates for the parameters $Rd,$ and ${\gamma }_{1}$ whereas it decays for the parameters $Pr$.
- The concentration of nanoparticles augments for the parameters $E, Nt$ and ${\gamma }_{2}$ but it falls short for the parameters $Nb, Le, {\sigma }^{*}$ and ${\lambda }_{C}$.
- The gyrotactic microorganisms boosted for the parameter ${\gamma }_{3}$ however, it reduces for the parameters $Lb$ and $Pe$.

The Galerkin-finite element method could be applied to a variety of physical and technical challenges in the future^72–83^. Some recent developments exploring the significance of the considered research domain are reported in the studies^84–93^.

## Data Availability

All data generated or analyzed during this study are included in this published article.

## List of symbols

$$\alpha$$: Viscoelastic parameter
$$E$$: Activation energy $$\left({\rm K}\,\, {\rm J}\,\, {\rm mol}^{-1}\right)$$
$\mathrm{H}$: Modified Hartman number
${\lambda }_{c}$: The relaxation time of mass (s)
${K}_{1} , {K}_{2}$: Homogeneous and heterogeneous reactions
$$T$$: Liquid temperature $$\left({\rm K}\right)$$
$$C$$: Liquid concentration $$\left({\rm kg}\,{\rm m}^{-3}\right)$$
$${T}_{\infty }$$: Ambient temperature $$\left({\rm K}\right)$$
$${C}_{\infty }$$: Ambient concentration $$\left({\rm kg}\,{\rm m}^{-3}\right)$$
${D}_{B}$: Coefficient of diffusion $\left({\rm m}^{2}\,{\rm s}^{-1}\right)$
$$q$$: Heat flux $$\left({\rm J}\,{ \rm m}^{-2}\,{\rm s}^{-1}\right)$$
$$J$$: Mass flux $$\left({\rm kg}\,{\rm m}^{-2}\,{\rm s}^{-1}\right)$$
$$\rho$$: Density $$({\rm kg}\, {\rm m}^{-3})$$
$$p$$: Pressure $$\left({\rm Kg}\,{\rm m}^{-1}\,{\rm s}^{-2}\right)$$
${C}_{p}$: Specific heat capacity $({\rm J}\, {\rm kg}^{-1}\, {\rm K}^{-1})$
$k$: Thermal conductivity of the liquid $({\rm W}\, {\rm m}^{-1}\, {\rm K}^{-1})$
